# Methods and Techniques to Facilitate the Development of *Clostridium novyi* NT as an Effective, Therapeutic Oncolytic Bacteria

**DOI:** 10.3389/fmicb.2021.624618

**Published:** 2021-03-29

**Authors:** Kaitlin M. Dailey, Reed I. Jacobson, Paige R. Johnson, Taylor J. Woolery, Jiha Kim, Rick J. Jansen, Sanku Mallik, Amanda E. Brooks

**Affiliations:** ^1^Cell and Molecular Biology Program, North Dakota State University, Fargo, ND, United States; ^2^Department of Pharmaceutical Sciences, North Dakota State University, Fargo, ND, United States; ^3^Department of Biological Sciences, North Dakota State University, Fargo, ND, United States; ^4^Department of Public Health, North Dakota State University, Fargo, ND, United States; ^5^Genomics and Bioinformatics Program, North Dakota State University, Fargo, ND, United States; ^6^Office of Research and Scholarly Activity, Rocky Vista University, Ivins, UT, United States

**Keywords:** CRISPR(clustered regularly interspaced short palindromic repeat)/Cas9(CRISPR associated protein 9)-mediated genome editing, oncolytic bacteria, *Clostridia*, gene engineering, oncotherapeutics

## Abstract

The tumor microenvironment is characterized by anomalous vascularization, hypoxia, and acidity at the core of solid tumors that culminates in concentrated necrosis and immune system dysregulation among other effects. While this environment presents several challenges for the development of oncotherapeutics that deliver their activity via the enhanced permeability and retention (EPR) effect of the leaky blood vessels around a tumor, oncolytic bacteria, or a class of bacteria with a noted capacity to lyse solid tumors, are attracted to the very environment found at the center of solid tumors that confounds other therapeutics. It is this capacity that allows for a potent, active penetration from the tumor margins into the core, and subsequent colonization to facilitate lysis and immune reactivation. *Clostridium novyi* in particular has recently shown great promise in preclinical and clinical trials when administered directly to the tumor. These studies indicate that *C. novyi* is uniquely poised to effectively accomplish the long sought after “holy grail” of oncotherapeutics: selective tumor localization via intravenous delivery. This study reports the development of efficient methods that facilitate experimental work and therapeutic translation of *C. novyi* including the ability to work with this obligate micro-anaerobe on the benchtop. Additionally, this study seeks to utilize this newfound experimental flexibility to address several gaps in the current knowledge regarding the efficacy of CRIPSR/Cas9-mediated gene insertion in this species to further develop this oncolytic bacteria and the genetic customization of bacteria in general.

## Introduction

Typically, the microenvironment of a solid tumor is considered a challenge for chemotherapeutic delivery. Characteristically poor vascularization of solid-state tumors is arguably the most difficult aspect of the microenvironment that limits the development of effective therapeutics. The disorganization of the intricate network of blood vessels caused by uncontrolled cellular growth leads to cells abnormally distant from local vessels, ultimately limiting oxygen diffusion as well as severely restricting other necessary nutrients (Minchinton and Tannock, 2006; McKeown, 2014). Furthermore, this aberrant vascularization is responsible for a build-up of metabolic byproducts carbonic and lactic acid—resulting in both hypoxic and acidic gradients, with highly concentrated conditions in the center of the solid tumor (Finger and Giaccia, 2010; McKeown, 2014). Additionally, these characteristics have a variety of impacts upon the environment surrounding the tumor, including suppressing the local immune system (Gonzalez et al., 2018; Boedtkjer and Pedersen, 2020). Recent literature reports have also indicated that this local tumor environment may intrinsically promote further tumor development and subsequent metastases (Boedtkjer and Pedersen, 2020). Despite these challenging conditions under which the majority of oncotherapeutics attempt to passively diffuse into the tumor with assistance from the enhanced permeability and retention (EPR) effect (Minchinton and Tannock, 2006), chemotherapeutics have been the standard of care for solid tumors for decades, and shown great efficacy (Miller et al., 2019). However, recent evidence has suggested certain chemotherapies may be responsible for inducing greater resistance and furthering aggressive metastases of the solid tumor (Redmond et al., 2008; Mansoori et al., 2017). A re-examination of oncolytic bacteria may circumvent some of these current challenges.

In direct contrast to the EPR mechanism of action, certain species of bacteria—collectively termed oncolytic bacteria—have an innate attraction to the type of environment found at the center of solid tumors, allowing for a potent, *active* migration to the hypoxic/acidic tumor core (Wei et al., 2008). Once the oncolytic bacteria have localized to the center of the tumor, they are able to successfully propagate and ultimately colonize and effectively influence the tumor (Diaz et al., 2005). Some select oncolytic bacterial species are able to directly lyse tumorigenic cells up to the normoxic margins of the tumor. Once they have migrated near the normoxic margin of the tumor, they can then re-activate and recruit the previously suppressed immune response to complete the tumor eradication (Wang et al., 2019). In order for current pharmaceutical therapeutics to accomplish both tumor lysis and immune system activation, typically more than one drug must be used, further complicating the development of effective therapeutics. While oncolytic bacteria are not a new discovery (Chien et al., 2017), due to several advantageous characteristics, it is unsurprising that they are reemerging on the therapeutic landscape (ClinicalTrials.gov., 2019).

**FIGURE 1:**
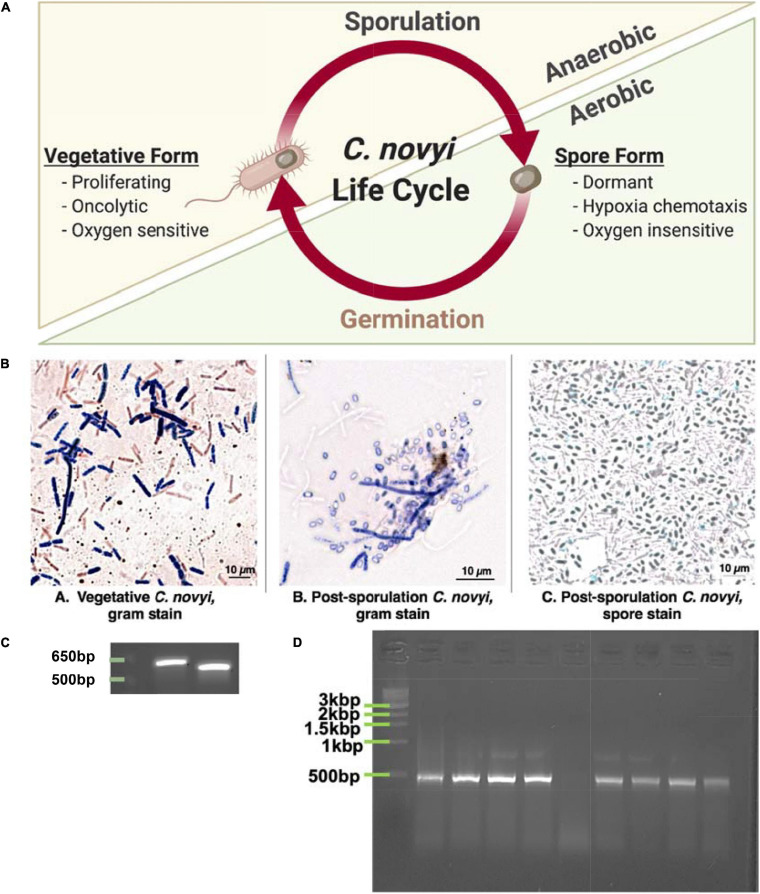
Demonstrating ability to grow *C. novyi.* **(A)** Schematic representation of *Clostridium novyi* life cycle. **(B)** Brightfield images captured under oil-immersion at 40×. From left to right: (a) Gram stain of vegetative *C. novyi*, (b) Gram stain of *C. novyi* post –sporulation, (c) Malachite green spore stain of post-sporulation *C. novy*i. **(C)** PCR amplicons primers designed with specificity to *C. novyi* 16s rRNA and α-toxin. **(D)** PCR amplification with *C. novyi* α-toxin primers after α-toxin knockout was performed.

In particular, the motile, gram-variable *Clostridium novyi* has demonstrated several beneficial innate characteristics that lend itself to development as an oncotherapeutic. This oncolytic bacterial species is one of the few capable of both direct and indirect oncolysis, as well as potent recruitment of the immune system due to its gram variability (Staedtke et al., 2016). *C. novyi* has the capacity to sporulate, resulting in a biphasic life cycle including: a proliferative, lytically capable vegetative form, and a more “dormant” sporulated form (Dang et al., 2001; Figure 1A). While the vegetative form is classified as an ultra-sensitive obligate anaerobe—and thus cannot survive in virtually any level of oxygen, the spore form does not show the same sensitivity (Figure 1A). In fact, sporulated *C. novyi* are able to survive atmospheric oxygen; however, the sporulated form of *C. novyi* cannot accomplish germination to the lytic vegetative form until an adequately hypoxic environment (such as the center of a solid tumor) has been located (Diaz et al., 2005; Staedtke et al., 2015, 2016). Unlike typical bacterial spores, *C. novyi* spores are thought to have some level of metabolic activity as they are able to sense and chemotax toward hypoxic/acidic gradients (Diaz et al., 2005), though the mechanism by which this occurs has yet to be elucidated. The culmination of these characteristics lends this particular oncolytic bacterial species to development as a potent therapeutic, which, at least in theory, has the capacity to treat not only a primary solid tumor, but also any metastases regardless of tissue location.

Indeed, *Clostridium novyi* has recently shown promise in mouse studies and preclinical trials (Wei et al., 2008; BioMed Valley Discoveries, Inc., 2019). Initial challenges of systemic toxicity encountered with *C. novyi* introduction to the bloodstream have been largely mitigated by the ability to create a non-toxic strain (*Clostridium novyi* NT) through a simple heat treatment causing the loss of the phage DNA encoded α-toxin responsible (Staedtke et al., 2016). Further studies detailing *C. novyi* NT introduction within murine models have suggested minimal toxicity and have observed no behavioral or histological signs of sepsis that were unable to be mitigated with the administration of fluids (Diaz et al., 2005). This landmark study also found that when *C. novyi* NT spores were intravenously delivered, 95% of murine subjects demonstrated some level of mitigation of subcutaneous tumors (Diaz et al., 2005). While this statistic is staggering in and of itself, upon examining the biodistribution of spores post-injection, it was shown that the vast majority of spores are quickly and innocuously cleared from subjects, with only around 1% of the initial dose localizing to the tumor (Diaz et al., 2005). Furthermore, as a testament to the exquisite specificity of this tumor-targeting effect, when other models of physiological hypoxia (i.e., ischemia) were tested, *C. novyi* NT colonization was not observed (Diaz et al., 2005). It is therefore reasonable to suggest that *C. novyi* NT spores could be modified for intravenous delivery to increase tumor localization.

In order to address these challenges and ultimately achieve clinical translation, a molecular toolkit must be developed through which to accomplish the modification of *C. novyi* NT spores, allowing them to “home” to a tumor upon intravenous injection. This study reports the development of efficient methods to facilitate experimental work and therapeutic translation of *C. novyi*. Additionally, it addresses several gaps in the current knowledge, and expands on the data regarding the efficacy of CRIPSR/Cas9 gene insertion in this particular species (Joseph et al., 2018).

## Materials and Methods

### Vegetative *C. novyi* Growth

*Clostridium novyi* used in this study were purchased from ATCC (19402) as a lyophilized powder. Two different methods were used to facilitate anaerobic growth: an atmospheric chamber and an oxygen-fixing enzyme.

#### Atmospheric Chamber

Reinforced Clostridial Media (RCM) powdered broth was prepared as per manufacturer’s instructions (38 g/1 L nanopure water). The RCM broth was autoclaved, and subsequently purged of oxygen through water bath pulse sonication for 90 min and sealed for immediate use within a benchtop atmospheric chamber (Glovebag—Spilfyter Hands-in-Bag 2-Hand Chamber). Purged media was aseptically aliquoted into sterile conical tubes within a sealed (Tyvek seam tape), carbon dioxide purged glovebag. Aliquots were inoculated with *C. novy*i which was maintained in anaerobicity by working exclusively within the glovebag. An oxygen indicator specifically developed for the microbiological culturing of anaerobic bacteria—which would turn blue to indicate a failure in the air-tight seal of the purged glovebag—was used to ensure anaerobiosis was not interrupted (OxyBlue Indicator, Oxyrase Inc.). Adequate biosafety was accomplished by purging the exhaust port through dual exposures to a sanitizer capable of mitigating spores (Spor-Klenz). Cultures were never exposed to oxygen as they were removed from the atmospheric chamber already sealed within airtight anaerobic chambers (BD GasPak EX container system) that included oxygen-fixing sachets (BD GasPakEZ sachets) to be placed in 37°C incubators without any agitation.

Solid RCM agar plate cultures were generated through the addition of 3% w/v agar (Sigma Aldrich) to RCM broth (BD Difco). The resulting solution was then autoclaved, poured into sterile petri plates aseptically in a Laminar flow hood, and allowed to solidify. Once solidified, plates were then degassed for 48 h prior to use by placing them in an anaerobic container with oxygen-fixing sachets. Again, plates were inoculated aseptically with *C. novyi* cultures within the sealed, purged glovebag and only removed once sealed within anaerobic containers to be transferred into a 37°C incubator.

#### Oxygen-Fixing Enzyme

In order to accomplish work with *C. novyi* outside of an atmospheric chamber, an enzyme capable of producing anaerobic conditions within bacterial media was used. It is important to note that this is still considered anaerobiosis through control of the microenvironment within the cultures themselves. Powdered RCM media was resuspended in nanopure water (38 g/L) and autoclaved for sterilization. Once the autoclaved media had cooled enough to be handled without autoclave gloves (∼45–48°C), Oxyrase for Broth (OB, Oxyrase Inc.) was added at 10% v/v aseptically within a Laminar flow hood. This solution of RCM/OB was incubated at room temperature in the hood for 30 min to allow the Oxyrase enzyme to fix the oxygen contained within the media. Then, the media was gently aliquoted to prevent the introduction of any excess oxygen into screw top conical tubes for immediate *C. novyi* inoculation using aseptic techniques within the hood. To ensure the continued anaerobicity of the enzyme-purged media, inoculated conicals were sealed with parafilm and placed within anaerobic containers. Because the Oxyrase enzyme will perpetually fix oxygen within the broth, it was not necessary to include oxygen-fixing sachets when using this method—the anaerobic containers were largely a biosafety measure to prevent cross contamination. As long as atmospheric oxygen was not reintroduced into these sealed cultures, an aerobic environment was maintained for at least a week. Resulting *C. novyi* cultures within containers were allowed to grow in a 37°C incubator without any agitation.

Solid agar plate cultures were prepared using powdered RCM media with 10% less water than manufacturer’s protocol (38 g/900 mL) with an added 3% v/v agar. This solution was then autoclaved to achieve sterility. Once the RCM agar solution had cooled enough to handle without autoclave gloves (∼45–48°C), 10% v/v Oxyrase for Agar (OA, Oxyrase Inc.) was added via gentle swirling to avoid foaming and thus introduction of more oxygen. Twenty-two milliliters of this solution was then pipetted aseptically into petri plates within a Laminar flow hood. These solid media cultures were then incubated in anaerobic chambers for bacterial growth (BD GasPak containers with sachets). Plates were sealed in airtight oxygen purged containers to solidify and used immediately. While this solid media is capable of fixing oxygen, due to the surface area exposed the endurance of the anaerobic conditions is limited to about 2 h of atmospheric exposure. Therefore, innoculated plates were incubated at 37°C in anaerobic containers with oxygen fixing sachets to minimized oxygen exposure and thus reserve the enzyme contained within the media for when the plates were being handled on the bench top.

#### *C. novyi* Freezer Stocks

*C. novyi* cultures were grown anaerobically as detailed previously at 37°C overnight. The resulting solution was spun at 13,000 rpm for 5 min to pellet vegetative cells and the supernatant was removed. The pellet was resuspended in RCM+10% Oxyrase for Broth+50% glycerol (v/v) as well as the erythromycin selective markers necessary for CRISPR plasmid retention when relevant. Resulting freezer stocks were stored at −80°C. Cultures remained viable for at least a year beyond generation.

#### Growth Curve

Vegetative *C. novyi* cultures were sub-cultured to an OD_600_ of 0.1, then allowed to grow for 74–96 h with 200 μL aliquots being harvested at 24 and 72 h to observe and record OD_600_.

### Sporulation of *C. novyi*

Again, two different methods were used to anaerobically force sporulation of vegetative *C. novyi* cultures: an atmospheric chamber and an oxygen-fixing enzyme.

#### Atmospheric Chamber

Sporulation media was adapted from previously published methods (Dang et al., 2001) and prepared as follows: 0.5 g Na_2_HPO_4_ (Sigma Aldrich), 3 g peptone (Fisher Scientific), 0.05 g L-cysteine (Alfa Aesar), 1 g maltose (Difco), per 100 mL nanopure distilled water. The resulting solution was brought to a pH of 7.5 with NaOH. After aliquoting the media (10 mL) into autoclavable, screw-top, glass jars, dried cooked meat particles (0.5% w/v, Difco) were added. Following autoclave sterilization, the media was degassed for 90 min in a sonicating water bath then sealed and transferred into a glovebag for immediate use. An aliquot of vegetative *C. novyi* cells in RCM media was inoculated into sporulation media within the oxygen purged glovebag. These cells were subsequently grown anaerobically in sealed anaerobic chambers with oxygen fixing-sachets (BD GasPak containers with sachets) for a week prior to spore isolation undisturbed.

#### Oxygen-Fixing Enzyme

Media was prepared as detailed in section “Atmospheric Chamber,” autoclaved and then aseptically aliquoted into screw-top conical tubes within a Laminar flow hood. Then, 10% v/v Oxyrase for Broth (Oxyrase Inc.) was added to facilitate oxygen-free sporulation media. Dried cooked meat particles were autoclaved separately, and 1–3 pellets (∼0.5% w/v) were added aseptically in a Laminar flow hood to each individual conical. An aliquot of vegetative *C. novyi* cells grown in RCM/OB as detailed previously in section “Oxygen-fixing Enzyme” was centrifuged at 2,000 rpm, and the media supernatant was removed from the cell pellet. Cells were gently resuspended in complete sporulation media to avoid the introduction of oxygen, and conical tubes were sealed with parafilm to prevent any further oxygen exposure that would deplete the OB efficacy. Again, because the Oxyrase enzyme will perpetually fix oxygen within the sporulation media, it was not necessary to include oxygen-fixing sachets, rather the anaerobic containers were largely to ensure biosafety and prevent cross-contamination with other bacterial species. These cultures were allowed to sporulate for a week undisturbed before isolation occurred.

#### Spore Isolation

The spore isolation protocol was adapted from a previously published protocol (Edwards and McBride, 2016) for purifying *C. difficile* spores. Briefly, the entire sporulation sample was centrifuged at 4,000 rpm in a swinging bucket rotor for 2 min at 4°C. The supernatant was removed into Spor-Klenz, and the pellet resuspend in sterile, ice cold distilled water and washed with sterile, ice cold distilled water three times, being pelleted by centrifugation in a fixed angle rotor at 13,000 rpm for 5 min each time. The washed spore preparation suspended in distilled water was then incubated at −20°C for at least 48 h to lyse any remaining vegetative cells. Samples were thawed and centrifuged at 13,000 rpm for 5 min, and the supernatant was removed. The resulting pellet was resuspended in 1 ml sterile, ice cold distilled water and further washed in ice cold distilled water as previously described for a total of five washes. At the conclusion of the washes, the spore preparation in 3 ml water was then gently added to the top of a 50% w/v sucrose gradient (10 mL, ACS grade, Research Products International) in a 15 ml polypropylene conical tube (Celltreat). The sucrose gradient was then centrifuged in a swinging bucket rotor at 4,000 rpm for 20 min at room temperature. The authors note that better results were achieved when the sucrose gradient was chilled, but not when centrifuged at 4°C (data not shown). Vegetative cells and debris subsequently collect at the interface and distribute throughout the gradient, while the spores form a small, white pellet at the bottom of the tube. After centrifugation, the cell debris and sucrose solution was carefully removed, leaving the spore pellet. This pellet was resuspended into 1 mL of sterile, room temperature distilled water and washed through repeated centrifugation at 13,000 rpm for 2 min in a fixed angle rotor for a total of five washes. The final spore preparation was resuspended in 1 mL purified distilled water.

#### Spore Activation

##### Heat

After sporulation and isolation, spores were forced to germinate to the vegetative state by heating the culture to 55°C in a heat block for 20 min as previously published (Edwards and McBride, 2016).

##### Tch Additives

As previously reported for *C. difficile* cultures (Edwards and McBride, 2016), a final concentration of 0.1% w/v sterile taurocholate (Tch, Sigma Aldrich) was added to the RCM/OB broth. Subsequently, spores were inoculated into Tch supplemented RCM/OB broth after heat activation.

#### Spore Enumeration

After spore activation, serial dilutions in sterile water were performed. 10 μL of each sterile dilution was spot plated onto RCM/OA agar plates and incubated anaerobically for 48 h. After 48 h, the plates were removed from the incubator and the colonies of each serial dilution spot were observed, counted, and recorded. The resulting number of colonies was used to calculate colony forming units (CFUs) per milliliter of media through this standard formula:

$$\frac{CFUs}{ml}\left(\#coloniesxdilutionfactor\right)/\left(volumeplated\right)$$

### Alpha-Toxin Knock Out

A previously published method for *C. novyi* alpha toxin knockout (Dang et al., 2001) was modified. Briefly, purified spores suspended in water were heated to 70°C for 15 min. Samples were then activated by a 20 min incubation at 55°C. Solid RCM/agar plates previously degassed for at least 48 h were then inoculated with 100 μL of treated spores per plate. Resulting colonies were allowed to grow for 48–72 h on RCM/agar in an anaerobic chamber at 37°C, then picked with a sterile tip to inoculate RCM broth within a glove bag. The subsequent liquid cultures were allowed to grow 48–72 h in an anaerobic chamber at 37°C. An aliquot was then harvested and concentrated to undergo colony PCR screening and confirm α-toxin knock-out.

### Colony PCR Screening

GoTaq Green PCR master mix (Promega) was added to the concentrated aliquot of *C. novyi* cells harvested in section “Alpha-Toxin Knock Out” along with the corresponding forward and reverse primers at a final concentration of 5 μM (*C. novyi* 16s rRNA fwd aagtcgtggctggctattt; *C. novyi* 16s rRNA rev ctccaagtgcctctccataag; *C. novyi* α-toxin fwd gattcaagaggccacagagatag; *C. novyi* α-toxin rev gacccaccttcaaaccactta, synthesized by IDT DNA) and adequate nuclease free H_2_O (Promega) to bring the final volume to 25 μL. Reactions were incubated in a thermocycler according to the following program: 95°C for 5 min (95°C 30 s; 45°C 60 s; 68°C 60 s)x40 cycles, 68°C 5 min. Resulting amplicons were loaded into a 1% agarose tris-buffered EDTA gel with ethidium bromide and separated at 120 V for 30 min and then imaged via an Aplengen Omega Lum G system.

### *C. novyi* Cell Staining

#### Gram Stain

An aliquot of *Clostridium novyi* liquid culture was placed on an un-coated glass microscopy slide (Fisher Scientific). The sample was heat fixed, then stained with crystal violet (BD Life Technologies) for 30 s, rinsed with distilled water, then soaked in iodine (BD Life Technologies) for a minute. Subsequently, the slide was decolored with 70% ethanol (BD Life Technologies), and counter stained with safarin red (BD Life Technologies) for a minute. Distilled water was used to rinse excess dye from the slide. The resulting sample was imaged via confocal microscopy (Zeiss Axio Imager M2) under oil immersion at 100×.

#### Spore Stain

After sporulation and spore isolation processes had been conducted, a small sample of the resulting solution was washed and resuspended in distilled water. This sample was then heat fixed to an uncoated glass slide for subsequent staining. 0.5% w/v malachite green (VWR) aqueous solution was used to cover the bacterial sample and slides were exposed to a steam bath for 5 min, adding more malachite green as necessary to prevent drying out. After 5 min the slide was rinsed with distilled water and counter stained with safarin red (BD Life Technologies) for a minute and washed again with distilled water. The resulting sample was imaged via confocal microscopy (Zeiss Axio Imager M2) under oil immersion at 100×.

### Comparison of *C. novyi* to *C. Difficile* and *E. coli*

The published *C. novyi* transcriptome (Bettegowda et al., 2007) was mined to determine the 10 highest and lowest expressed genes. These gene sequences were then used to identify putative orthologous genes in both *Escherichia coli* and *Clostridium difficile* through utilization of NCBI BLASTn, UniProt, and GeneID databases. While orthologous sequences for all genes were not located—especially those for the 10 least expressed *C. novyi* genes, which are presumably species specific—those that were identified were used to generate a codon adaption index (CAI) as well as the gene characteristics of GC skew and percent purines (%R) through previously published formulas (Sharp and Li, 1987; Musto et al., 2003). Putative molecular fraction (% mol) values were mined from previously published gene expression data (Ishihama et al., 2008; Janoir et al., 2013; Permpoonpattana et al., 2013) and compared to establish the similarity of *C. novyi* to both *C. difficile* and *E. coli*—more well characterized bacteria species.

### CRISPR/Cas Plasmid Design

#### Cas Enzyme

pNICKclos1.0 (Addgene #73639), an engineered CRISPR/Cas9 plasmid, was purchased and used for these experiments. This plasmid contained sequences encoding *Streptococcus pyogenes* Cas9nickase protein (5′-NGG-3′ protospacer adjacent motif) as well as multiple cloning sites including the *Spe*I/*Not*I sites for crRNA insertion and *Not*I/*Xho*I for the gene insert. This plasmid has been used and published to successfully perform CRISPR-mediated gene editing in *Clostridium* species previously (Joseph et al., 2018). For clarification, Figure 4B contains a detailed schematic of the cloning cassette inserted into this plasmid backbone.

#### Gene Insertion Target Selection

Data was mined from the transcriptome tables produced (Bettegowda et al., 2007) to identify an appropriate target for gene integration into the spore’s coat for ease of identification and assessment. Gene targets were assessed by the following criteria; (1) integration of the gene insert under the promoter for a surface display protein, (2) avoidance of genes encoding chemotaxis or anerobic functions, including the operon containing NT01CX2374, NT01CX2375, NT01CX2376, (3) avoidance of any lipases NT01CX0979, NT01CX2047, and NT01CX0630, (4) avoidance of spore genes highly upregulated during tumor infection (21 genes identified (Bettegowda et al., 2007). Based on this study’s expression goal, four genes (NT01C0401, NT01CX0481, NT01CX1621, and NT01CX1736) were identified as appropriate targets utilizing these criteria.

#### sgRNA Design

The primary nucleotide sequence for these four genes was manually analyzed for protospacer adjacent motif (PAM) sequences correlating to the SpCas9n contained within pNICKclos1.0. Once candidate sequences were generated, ThermoFisher^1^ as well as IDT DNA CRISPR/Cas9 design tools^2^ were used to compare the CRISPR RNA (crRNA) sequence options generated. crRNA sequences were ranked by on-target efficacy, off-target potential (as determined manually via a BLASTn search of the *C. novyi* genome for sequences with high percent identity) and secondary structural concerns (*e.g.*, to avoid internal hairpin formation). Two crRNA sequences were chosen for each gene to be synthesized as gene blocks from Invitrogen. The primary sequence of the crRNAs were synthesized within cloning restriction enzyme sites correlating to the cloning sites present on pNICKclos1.0 (*Spe*I and *Not*I) with an additional unique restriction digest site (*Bgl*II) included for use in cloning verification. The trans-activating CRISPR RNA (tracrRNA) necessary to recruit *Sp*Cas9n was included in the backbone immediately to the 3’ of the cloning restriction digest sites and was not modified from the original sequence contained within pNICKclos1.0.

#### Flanking HDR Arm Design

The gene sequence 1 kbp upstream and 1 kbp downstream of the cleavage site determined by the selected crRNA sequenced was used to generate the homologous arms that flank the gene insert. Both arms were contained within unique restriction digest cloning sites (*Not*I-*Kpn*I for the upstream arm and *Sac*I-*Xho*I for the downstream arm) to lend unique flexibility to this CRISPR cassette (Figure 4B). In order to accomplish validation through the construction and cloning of this plasmid, a unique verification restriction digest site, *Kpn*I, was included.

#### Gene Insert

A simple six amino acid sequence was modified to account for *C. novyi* codon bias. This sequence was chosen as a proof-of-concept to test if CRISPR-mediated genome modification can occur in *C. novyi*. A Shine-Dalgarno sequence and TATA box were included upstream of the gene insert before the start codon, also translated to account for codon bias. Additionally, an *EcoR*V enzyme site was designed to be present within the HDR template to be used for repair. This restriction digest site would then be incorporated into the genome, indicating gene modification was successful.

### CRISPR/Cas Plasmid Construction

#### crRNA and HDR Cassette Synthesis

HDR arms were synthesized to contain an eighteen-nucleotide gene insert between the two flanking arms as a single cassette for ease of cloning. Both the HDR cassette and correlating crRNA sequence were synthesized by GeneArt Gene Services (Thermo Fisher Scientific).

#### crRNA Insertion

The resulting oligonucleotide from GeneArt and the pNICKclos1.0 plasmid was digested with *Spe*I-HF and *Not*I-HF (New England Biolabs, Inc.) enzymes in CutSmart Buffer (New England Biolabs, Inc.) at 37°C for 1 h to generate coordinating sticky-end overhangs. These samples were then run out on a 3% agarose gel at a low voltage, and gel excision was used to isolate the correct fragments. Subsequently, fragments were purified via the GeneJET Gel Extraction Kit (Thermo Fisher Scientific). Purified fragments were then combined with 10 μL NEBuilder HiFi DNA Assembly Master Mix (New England Biolabs, Inc.) in a 2:1 insert to vector ratio for a total volume of 20 μL. This ligation mixture was incubated at 50°C for an hour, then transformed into *E. coli* immediately. NEB 5-alpha Chemically Competent *E. coli* (New England Biolabs, Inc.) were transformed via manufacturer’s protocol (30 min incubation of 2 μg plasmid with 25 μL of cells for 30 min, 90 s heat shock at 42°C, returned to ice for 2 min, then resuspended in 500 μL of SOC media, incubated in shaking incubator for 1 h with 100 μL plated on ampicillin containing media) with the assembled plasmid. Candidate colonies were grown up in Luria Broth culture supplemented with ampicillin as a selective marker. Transformed plasmid DNA was isolated via GeneJET Plasmid Miniprep kit (Thermo Fisher Scientific) and the resulting plasmid DNA was digested with *Bgl*II-HF in CutSmart Buffer at 37°C for 1 h. Digested DNA was run on a 1% agarose gel and analyzed to verify the crRNA sequence had been inserted into pNICKclos1.0. Once crRNA insertion was confirmed, the pNICKclos1.0+sgRNA plasmid was used for HDR cassette insertion.

#### HDR Cassette Insertion

The purchased gene cassette from GeneArt and the pNICKclos1.0+sgRNA plasmid were digested with *NotI*-HF and *Xho*I (New England Biolabs, Inc.) enzymes in CutSmart Buffer (New England Biolabs, Inc.) at 37°C for 3 h to generate coordinating sticky-end overhangs. These samples were then run out on a 3% agarose gel at a low voltage, and the correct fragments were excised from the gel. Excised fragments subsequently underwent gel purification via the GeneJET Gel Extraction Kit (Thermo Fisher Scientific). Purified fragments were then ligated using NEBuilder HiFi DNA Assembly Master Mix (New England Biolabs, Inc.) in a 2:1 insert to vector ratio for a total volume of 20 μL. This ligation mixture was incubated at 50°C overnight out of direct light. The reassembled, engineered plasmid was then transformed into NEB 5-alpha Chemically Competent *E. coli* (New England Biolabs, Inc.) 30 min incubation of 2 μg plasmid with 25 μL of cells for 30 min, 90 s heat shock at 42°C, returned to ice for 2 min, then resuspended in 500 μL of SOC media, incubated in shaking incubator for 1 h with 100 μL plated on amplicillin containing media, with ensuing candidate colonies grown up in Luria Broth cultures with ampicillin and harvested via GeneJET Plasmid Miniprep isolation kit (Thermo Fisher Scientific). The resulting plasmid DNA was digested with *Kpn*I-HF in CutSmart Buffer at 37°C for 1 h. After incubation, the digestion was run out on a 1% agarose gel and analyzed to verify insertion of the HDR cassette into pNICKclos1.0+sgRNA002. Once HDR cassette insertion was confirmed, the complete pKMD002 plasmid was transformed into NEB 5-alpha Chemically Competent *E. coli* (New England Biolabs, Inc.) by the protocol detailed previously and a GeneJET Maxiprep plasmid isolation kit (Thermo Fisher Scientific) was used to harvest and purify a stock of plasmid DNA. This purified plasmid DNA was used to transform calcium competent *C. novyi*.

### Preparation of Calcium Competent *C. novyi*

RCM/OB broth was inoculated with vegetative *C. novyi* and incubated at 37°C in anaerobic conditions overnight. A 30 mL of RCM/OB was inoculated to contain a final concentration of 10% v/v of the previous overnight culture. This larger culture was allowed to grow at 37°C in anaerobic conditions overnight to a desired OD_600_ of 0.6–0.8. The authors noted that while ODs above this range were largely indifferent, ODs below this range had difficulty surviving the protocol (data not shown). The resulting culture was moved to conicals prechilled at −20°C overnight and centrifuged at 2,000 rpm and 4°C for 40 min. The resulting supernatant was removed, and the pellet was resuspended in 8 mL 0.1 M CaCl_2_ (EMD Millipore) + 10% v/v Oxyrase for Broth (Oxyrase Inc.) prechilled to 4°C. This solution was incubated on ice for 30 min, then centrifuged in a swinging bucket rotor at 2,000 rpm and 4°C for 40 min. The supernatant was removed and the pellet was resuspended in 2 mL prechilled (4°C) 0.1 M CaCl_2_:15% glycerol (Thermo Fisher Scientific) + 10% v/v Oxyrase for Broth, then aliquoted into prechilled (−20°C) Eppendorf tubes.

#### Transformation of Calcium Competent *C. novyi*

Calcium competent *C. novyi* cells were allowed to thaw on ice. Subsequently, 5 μg of either purified pUC19 control plasmid (New England BioLabs Inc.) or the purified pKMD002 plasmid was added to an empty prechilled 15 mL tube (4°C). Competent *C. novyi* cells (100 μL) were added directly on top of the plasmid DNA in the prechilled tube without vortexing or mixing. The resulting solution of cells and plasmid DNA was incubated on ice for 30 min. This mixture was then heat shocked at exactly 42°C for precisely 90 s and immediately returned to ice for 2 min. RCM/OB broth was added to each sample, which was then allowed to grow over night at 37°C anaerobically. Note that 24 h of growth is the approximate equivalent to a single life cycle for *C. novyi*, and thus the selective marker was not added until adequate time was allowed for plasmid uptake and expression. After 24 h, ampicillin (50 mg/ml final concentration, Fisher Scientific) was added to cells transformed with pUC19 control plasmid while erythromycin (250 μg/ml final concentration, Sigma Aldrich) was added to cells transformed with pKMD002. Regardless of the antibiotic selective pressure applied, all transformed cells subsequently were allowed to grow anaerobically for 24 h at 37°C. Cells were then plated onto RCM/OA/ampicillin (*C. novyi* transformed with pUC19) or RCM/OA/erythromycin (*C. novyi* transformed with pKMD002) agar plates and grown in OxyPLUS anaerobic chamber plates for 48 h. Resulting colonies were counted and used to calculate colony forming units per milliliter media of the successfully transformed *C. novyi*.

### Verifying Plasmid Transformation

Candidate colonies resulting from the pKMD002 transformation were picked, designated Candidates A–E, and grown in RCM/OB broth for 48 h. Selective marker pressure was not maintained beyond 48 h to facilitate plasmid loss, preventing off-target CRISPR DNA breakage and cell stress that would result in cell death. An aliquot was harvested during the 48 h selective pressure period to determine if the pKMD002 plasmid was indeed present after transformation. This aliquot of *C. novyi* underwent plasmid DNA isolation via GeneJET Plasmid Miniprep kit (Thermo Fisher Scientific). PCR utilizing GoTaq Green PCR MasterMix (Promega) and primers specific to the HDR domain of pKMD002 (primers: Internal HDR forward—tttactcagccttaggatttacaga, Internal HDR reverse—tcaggtatagttgcaggaatgaa) was done with isolated plasmid as the template, according to the following program: 95°C for 5 min (95°C for 30 s, 45°C for 1 min, 72°C for 2 min) × 40 cycles, and then 72°C for 5 min. The resulting amplicons were restriction digested with *EcoR*V-HF (New England BioLabs, Inc.) in CutSmart Buffer at 37°C for 1 h, then run out in a 1% agarose gel at 120 V. Gels were imaged on an Aplegen Omega Lum G gel imaging system and analyzed to verify the presence of pKMD002. Figure 5A has been included for clarity of the methods used for validating gene modification in this study.

### Verifying Genomic Modification

Aliquots of Candidates A–E were harvested after 48 h of growth in non-selective media. These samples underwent TRIzol (Zymo Research) genomic DNA isolation according to manufacturer’s protocol. Extracted genomic DNA then underwent PCR utilizing primers specific to the HDR domain contained within pKMD002 (see section “Verifying Plasmid Transformation”) that by design include a unique *EcoR*V restriction site for verification. PCR was performed using GoTaq Green PCR MasterMix (Promega) and a thermocycler program as follows: 95°C for 5 min (95°C for 30 s, 45°C for 1 min, 72°C for 2 min) × 40 cycles, 72°C for 5 min. The resulting amplicons were digested with the *EcoR*V-HF (New England BioLabs, Inc.) in CutSmart Buffer at 37°C for 1 h and run on a 1% agarose gel at 120 V. Gels were imaged and analyzed for genomic insertion after exposure to pKMD002 as previously described. Again, Figure 5A has been included for clarity of the methods described here.

### Determining Off-Target Effects

#### Growth Curves

RCM/OB cultures were grown for Candidates A–E and sub-cultured to an OD_600_ of 0.1, then allowed to grow for 74–96 h with aliquots being harvested at as previously described to establish growth curves.

#### Spore Enumeration

Candidates A-E underwent forced sporulation and germination as detailed previously. Colony forming units were observed and recorded to determine CFU/mls.

#### Cell Lysis

PANC-1 cells were grown using Dubelco’s Modified Essential Media (DMEM, Caisson Labs) with 10% v/v fetal bovine serum (VWR) and 1% v/v penicillin-streptomycin (Caisson Labs) and 1% v/v fungicide additives (Fungizome Antimycotic, Thermo Fisher Scientific). Cells were plated at 100,000 cells per well in a 12-well plate (Corning Costar) and were allowed to attach at 37°C in 5% carbon dioxide overnight. Subsequently, adhered cell cultures were inoculated with *C. novyi* cultures at a concentration of 5,000 spores per well as determined by spore enumeration detailed in section “Spore Enumeration.” Co-cultures of human and bacterial cells were incubated both aerobically (normal cell culture) and anaerobically (in BD GasPakEZ containers with sachets) at 37°C for 24 h. The media was then removed, and wells were rinsed with phosphate buffered saline (Caisson Labs) before fresh media containing purified resazurin (44 μM final concentration, Thermo Fisher Scientific) was added. Cells were incubated with the cell viability determining enzyme for 5 h, and then Abs_570_ was observed and recorded.

## Results

### Establishing Growth of *C. novyi* (Figure 1)

#### *C. novyi* Cell Images

Since *C. novyi* is an ultrasensitive anaerobe when in its vegetative, proliferative state even growing this microbe prior to any manipulation was challenging. Initially, cells were grown in RCM media within a bench top atmospheric chamber (a glovebag). Maintaining an anaerobic atmosphere under these conditions can be challenging; hence, to confirm the growth of vegetative *C. novyi* (ATCC 19402), cells were gram stained. A gram-stained sample of vegetative *C. novyi* visually confirmed the presence of gram-variable rod-shaped bacteria. Alternatively, vegetative cells were forced to sporulate as described in the methods, and spores were stained with malachite green. Similarly, to ensure that spores had indeed been isolated, cells were forced to germinate through a modified heat treatment protocol and stained with gram stain (Figure 1Bb) as well as a malachite green differential stain. The presence of spores was visually confirmed (Figure 1Bc). Although cultures visually appeared and stained as expected for *C. novyi*, 16s rRNA PCR was used to confirm the genetic identity of the microbe phenotypically presenting as *C. novyi*. The *in silico* predicted amplicon sizes for species specific primers for *C. novyi* 16s rRNA and α-toxin were 578 and 533 bp, respectively. Colony cracking and PCR of the anaerobic bacterial cultures resulted in amplicons of the expected length (Figure 1C), confirming the presence of *C. novyi* specifically.

#### α-Toxin Removal

Establishing a Non-Toxic strain of *C. novyi* (*C. novyi* NT) required the removal of a phage DNA plasmid contained within Wild Type *C. novyi*. A modification of the knock-out published heat treatment protocols (Dang et al., 2001) was performed on sporulated *C. novyi* cells to include an activation step at 50°C. Briefly, PCR with species specific primers for *C. novyi* α-toxin was conducted to confirm the absence of a 533 bp amplicon that results from α-toxin phage DNA, thus confirming this protocol adaptation had yielded several *C. novyi* NT colonies (Figure 1D).

### Comparison of Growth Techniques (Figure 2)

**FIGURE 2:**
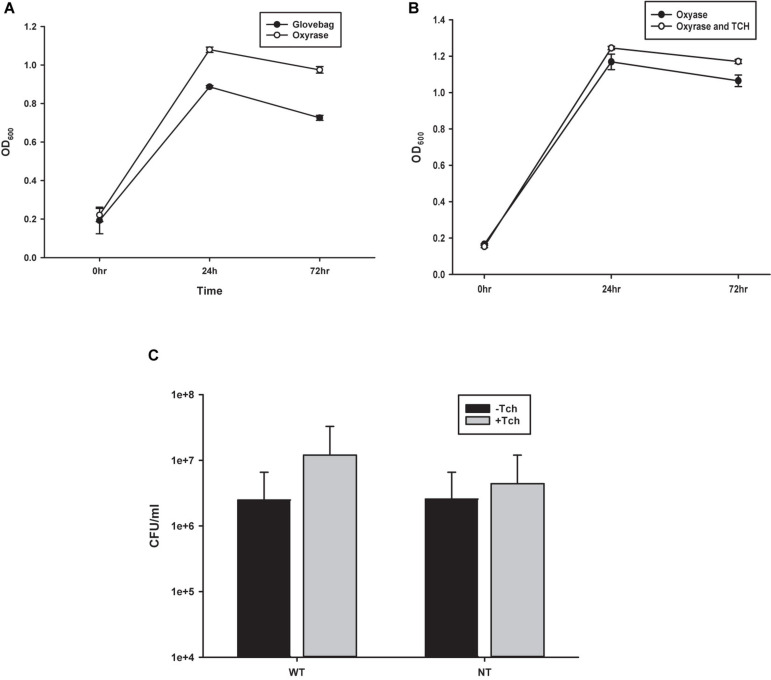
Comparison of growth techniques. **(A)** Growth curves from observing OD_600_ of *Costridium novyi* under glovebag conditions vs. with oxyrase enzyme. No statistical difference was found at any time point as determined by a Student’s standard *t*-test (*n* = 3 samples per time point). **(B)** Growth curves from observing OD_600_ of *Costridium novyi* under in oxyrase vs. oxyrase with the addition of taurocholate. No statistical difference was found at any time point as determined by a Student’s standard *t*-test (*n* = 3 samples per time point). **(C)** Spore enumeration of *Clostridium novyi* wild type and non-toxic strains with and without the addition of taurocholate. No statistical difference was found at any time point as determined by a Holms-Sidak test (*n* = 3 samples per enumeration).

To probe the efficacy of the oxygen-fixing microbial broth additive Oxyrase for Broth (OB) to create an anaerobic environment capable of sustaining *C. novyi* cells, *C. novyi* vegetative cells were seeded in both a carbon dioxide purged atmospheric chamber (glovebag) and in an aerobic atmosphere with broth containing OB. Aliquots were removed every 24 h over 72 total hours and OD_600_ was observed and recorded (Figure 2A).

#### Addition of Tch

Previous publications describing methods for *C. difficile* have indicated that higher levels of spore germination to vegetative cells can be achieved with the addition of 0.1% taurocholate (Tch) to the media (Edwards and McBride, 2016). To establish the effect of supplementing the media with Tch, as well as begin to probe the efficacy of using already established *Clostridia* protocols with *C. novyi*, growth curves were generated. Purified *C. novyi* spores were forced to germinate through heat activation in RCM/OB media or RCM/OB/Tch media, and resulting cultures were observed by OD_600_ for 72 h (Figure 2B). Notably, previous experiments comparing RCM with RCM plus Oxyrase for broth indicated that there was no significant difference in growth due to Oxyrase (Figure 1A). The resulting growth curves demonstrated no statistical significance when Tch was added to the media. To further corroborate these results, spores were isolated and subsequently forced to germinate, and ensuing spore enumeration was quantified via serially diluted colony forming units on solid RCM/Oxyrase for Agar (OA) media. Again, no statistical difference was observed for either *C. novyi* Wild Type or Non-Toxic strain with the addition of Tch to the media.

### CRISPR/Cas9n Plasmid Design (Figure 3)

#### Comparing *C. novyi* Genes to *C. Difficile* and *E. coli*

**FIGURE 3:**
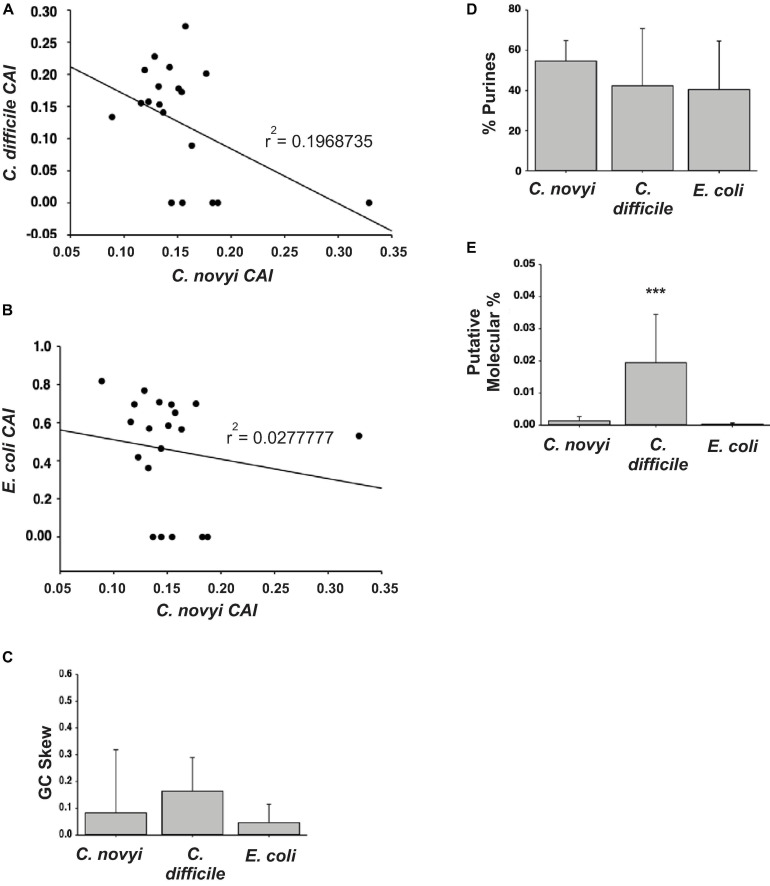
Designing the plasmid. **(A,B)** The resulting codon adaption index (CAI) values for orthologous genes in *C. difficile* or *E. coli* were plotted against those of *C. novyi* to ascertain a correlation. **(C,D)** Upon the comparison of the primary sequences of *C. novyi* genes with the orthologs found in *C. difficile* and *E. colie*, the GC skew **(C)** was determined as was the percent purines for each sequence **(D)**. **(E)** The literature was mined for expression data and the putative molecular percent was obtained for each gene and its ortholog. ^∗∗∗^indicates a statistical significance of *p* < 0.001.

In order to select the most suitable targets for insertion into the genome, it was necessary to determine the relationship of gene sequences of *C. novyi* compared to those in the more characterized species, *C. difficile* and *E. coli*. The 10 most and 10 least expressed genes from the published transcriptome (Bettegowda et al., 2007) were compared to orthologous sequences found through BLASTn searches in *E. coli* and *C. difficile*. The codon adaption index (CAI) was then determined through the application of a well-known formula (Sharp and Li, 1987; Musto et al., 2003), and the resulting CAI were used to create a scatter plot to establish the correlation between *C. novyi* and *C. difficile* as well as *C. novyi* and *E. coli* (Figures 3A,B). Basic characteristics, such as the GC skew (Figure 3C), percent purines (%R, Figure 3D) of *C. novyi* genes and putative orthologs were determined based on the primary sequence for each gene. Additionally, a putative molecular percentage of total protein expression for the protein encoded by each gene was mined from the literature (Ishihama et al., 2008; Janoir et al., 2013; Permpoonpattana et al., 2013) and compared in Figure 3E. No significant correlation was found for the comparative analysis of gene sequences, indicating a necessity to manually design and implement experimental methods and gene constructs specific to *C. novyi*.

After mining the published *C. novyi* transcripts and proposed proteome (Bettegowda et al., 2007), several genes were chosen as targets within which to insert a foreign gene encoding a six amino acid tag. Targets were selected ultimately based on the following criteria: (1) integration of the gene insert under the promoter for a surface display protein, (2) avoidance of genes encoding chemotaxis or anerobic functions, including the operon containing NT01CX2374, NT01CX2375, NT01CX2376, (3) avoidance of lipases NT01CX0979, NT01CX2047, and NT01CX0630, (4) avoidance of spore genes highly upregulated during tumor infection (21 genes identified; Bettegowda et al., 2007). Expression of the tag was thus targeted to the surface of the spore coat. Subsequent sgRNA and HDR gene cassettes were designed with these four genes as targets: NT01C0401, NT01CX0481, NT01CX1621, and NT01CX1736 (Table 1).

**TABLE 1:** Table indicating the chosen *C. novyi* genes to target CRISPR mediated gene insertion and a few of the important characteristics considered when selecting these targets.

Candidate Gene ID	Unique to *C. novyi*?	Predicted Functional Class	Predicted Function within class	Predicted to be extracellular?	Spore mRNA abundance	Upregulated during infection?
NT01CX0401	Yes	Cell envelope	Other	Yes	++	++
NT01CX0481	Yes	Cell envelope	Other	Yes	+	+
NT01CX1621	No	Cell envelope	Biosynthesis of murein sacculus and peptidoglycan	Yes	+	+
NT01CX1736	No	Cell envelope	Biosynthesis and degradation of surface polysaccharides and lipopolysaccharides	Yes	+	+

*Data synthesized from Bettegowda et al. (2007).*

### Building the CRISPR/Cas9n Plasmid (Figure 4)

**FIGURE 4:**
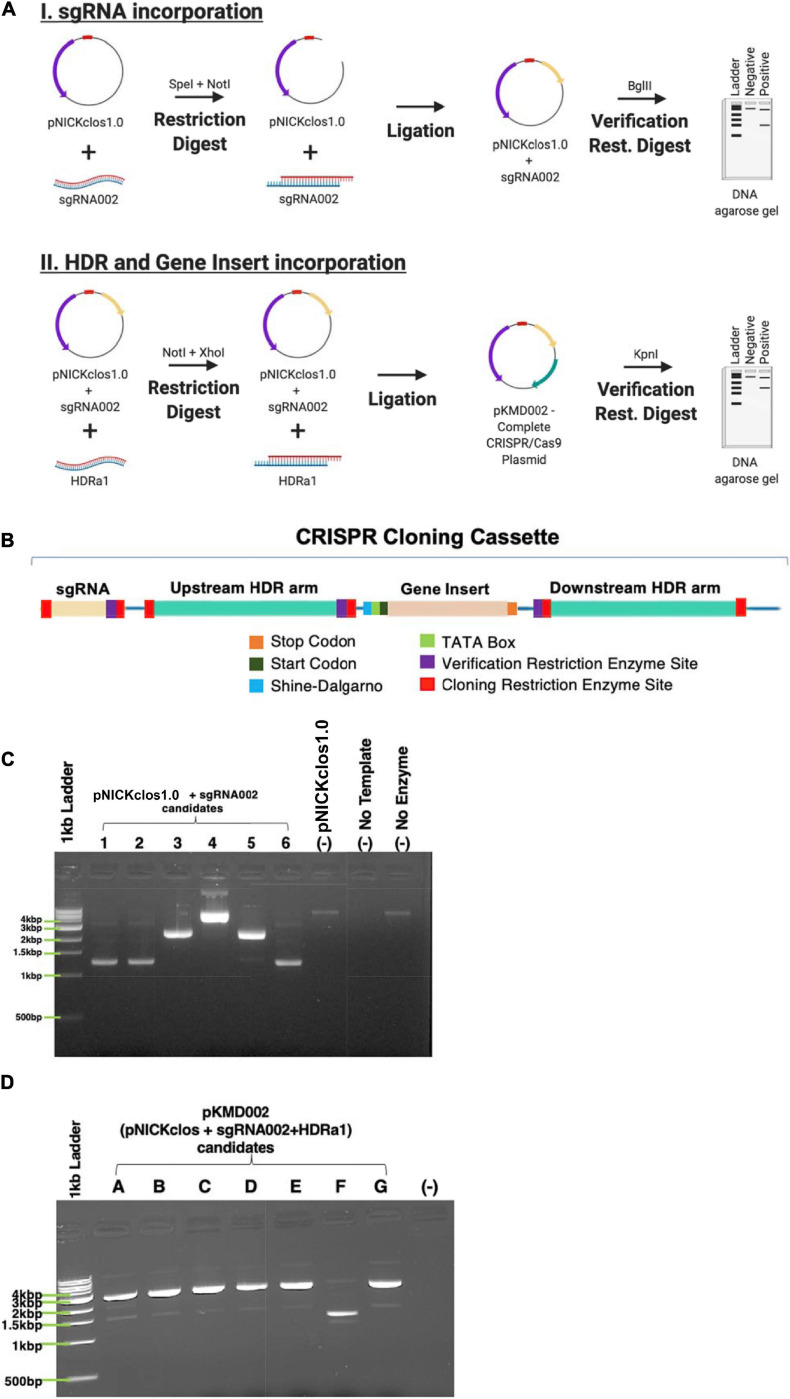
Building the CRISPR/Cas9n plasmid. **(A)** Schematic representation of stepwise cloning method. **(B)** DNA domain map of CRISPR cloning cassette build and utilized within pNICKclos1.0 to generated plasmid (pKMD002) used in this study. **(C)** Verification restriction digests confirming the insert of sgRNA002 targeting NT01CX0401. Singlet is negative for insertion of desired sgRNA after digest with BglII, doublet indicates positive insertion. **(D)** Verification restriction digests confirming the insert of HDR cassette corresponding sgRNA002 to gene target NT01CX0401. Singlet is negative for insertion of desired sgRNA after digest with KpnI, doublet indicates positive insertion.

The CRISPR cloning cassette (Figure 4B) to insert the gene encoding a six amino acid tag was built in the pNICKclos 1.0 backbone. To facilitate downstream screening as well as to build a versatile cloning system, several restriction sites were included. Briefly, the *Bgl*II restriction digest site was predicted by *in silico* experiments to indicate successful insertion of the desired sgRNA sequence by resulting in a doublet of 10.1 kb and 973 bp. If cloning was unsuccessful, *in silico* digestion resulted in a single band of 11 kb after digestion. Using this strategy, several candidate plasmids were observed to produce the correctly sized doublet pattern, indicating successful insertion of sgRNA002 into the pNICKclos1.0 backbone (Figure 4C). pNICKclos 1.0 with the inserted sgRNA002 was subsequently used to integrate the HDR cassette. Once again, *in silico* modeling indicated successful insertion of the entire HDR and gene insert cassette would be indicated by doublet bands at 8.137 and 2.967 kb after digestion with the validation enzyme *Kpn*I, while unsuccessful cloning would result in a single fragment of 11 kb. Using this strategy, *Kpn*I digestion resulted in several candidates, completing the CRISPR/Cas9n plasmid pKMD002 (Figure 4D).

**FIGURE 5:**
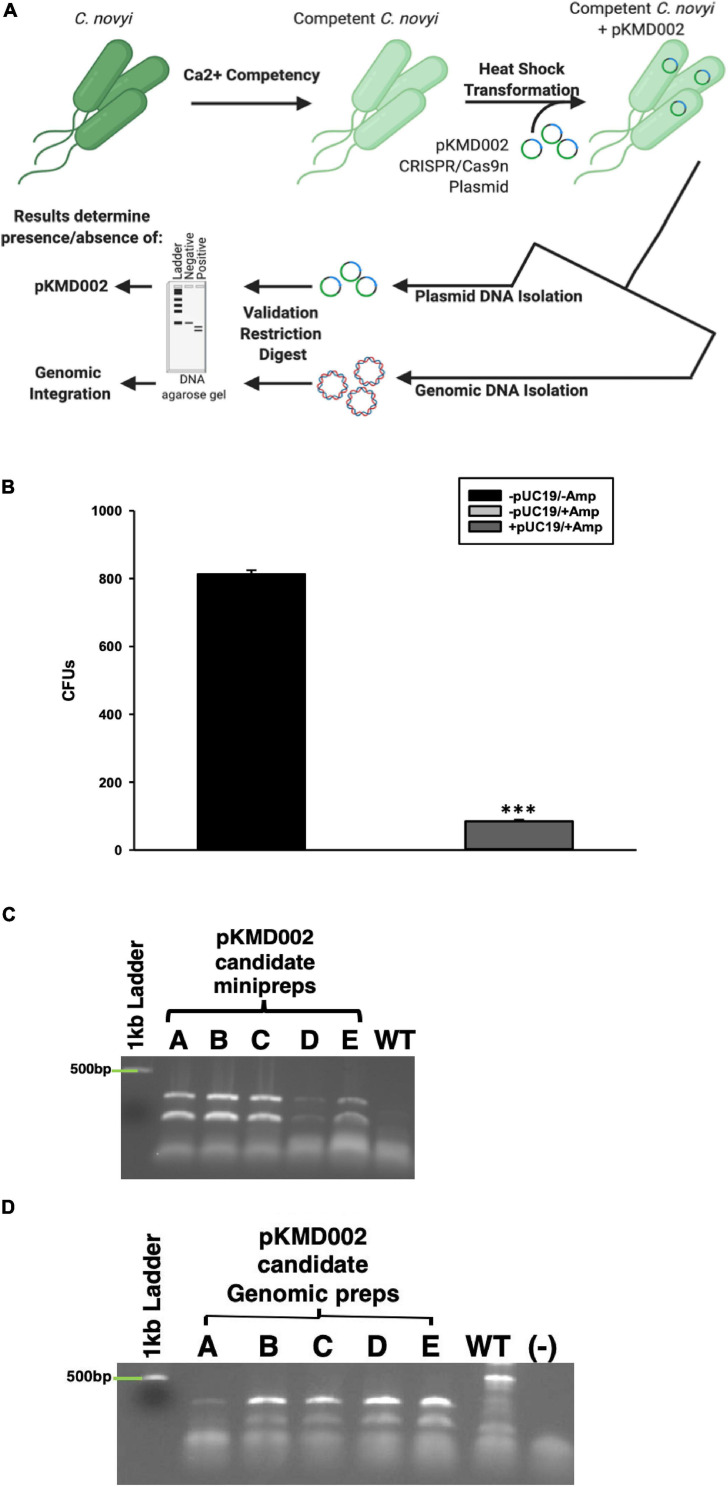
Calcium Competent *C. novyi* Transformations. **(A)** Schematic representation of experimental flow. **(B)** Resulting colony forming units after *Clostridium novyi* cells underwent calcium competent transformation with the *E. coli* plasmid pUC19, which contains a gene encoding ampicillin resistance. Statistical significance was determined to be *p* < 0.001 through the application of the Holms-Sidak test (*n* = 3 experiments). **(C)** After calcium competent *C. novyi* were transformed with pKMD002, five candidates (A–E) underwent plasmid minipreperation, and subsequent restriction digest to confirm plasmid present in cultures. Singlet is negative for presence, doublet is positive due to the presence of a *EcoR*V site designed in the insert. **(D)** After calcium competent *C. novyi* were transformed with pKMD002, five candidates (A–E) underwent genomic DNA isolation, and subsequent restriction digestion to confirm plasmid present in cultures. Singlet is negative for genomic insertion, doublet is positive due to the presence of a *EcoR*V site designed within the insert. ^∗∗∗^indicates a statistical significance of *p* < 0.001.

Earlier attempts to transform *Clostridia* sp.s, or indeed virtually any species other than *E. coli*, have met with extremely low efficiency plasmid uptake. To overcome this challenge, a standard protocol to create calcium competent *E. coli* was modified to include Oxyrase enzymes for use with *C. novyi*. To confirm the validity of this method, prepared competent *C. novyi* were transformed with pUC19. The resulting colonies after transformation were counted and colony forming units per milliliter of PBS was determined (Figure 5B). A significant difference was observed for transformations that occurred without ampicillin or pUC19 (*p* < 0.001). Subsequently, calcium competent *C. novyi* cells were transformed with the complete CRISPR/Cas9 plasmid, pKMD002, and grown under erythromycin selective pressure. No breakthrough colonies were noted. Several colonies grew on erythromycin containing RCM/OA plates after transformation with pKMD002, indicating several candidates for CRISPR/Cas9 gene modification. Upon isolating plasmid DNA from the candidate colonies, PCR was conducted with primers corresponding to the HDR arms and amplicons were validated via restriction digest with *EcoR*V. All five tested candidates contained the pKMD002 plasmid as per the doublet pattern observed (Figure 5C). Once the presence of the plasmid was confirmed, genomic DNA was isolated to evaluate if introduction of pKMD002 had conferred a genomic insertion of the cassette encoding the six amino acid tag. Primers correlating to the sequence flanking the site of gene insertion were used with genomic DNA from each candidate as a template (Figure 5D). *EcoR*V was used to digest the amplicons, with those candidates positive for gene insertion showing a doublet after *EcoR*V digestion, indicating that the HDR template had been used to repair the DNA damaged by Cas9n. This doublet appeared in all five candidates as predicted and was absent in un-transformed *C. novyi* genomic DNA. Thus, five positive clones were identified after transformation with pKMD002.

**FIGURE 6:**
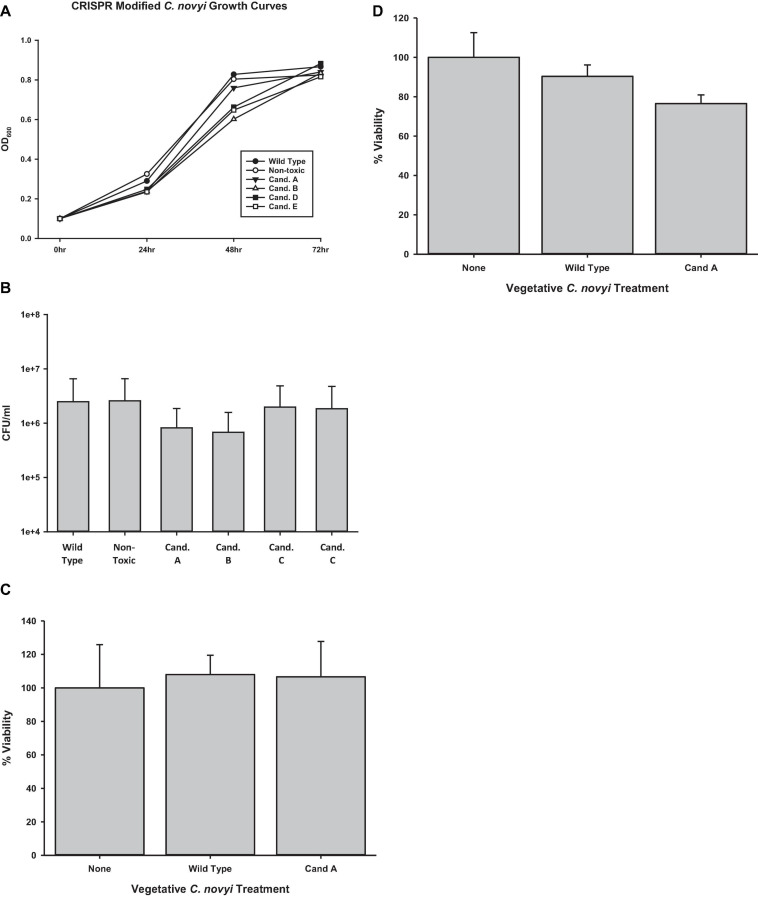
Determining off-target effects of CRISPR/Cas9 mediated gene editing. **(A)** Growth curves were determined by observing candidates A–E were for 72 h collecting OD600 at significant time points. No statistical difference was determined at any time point (*n* = 3 for each time point, Holms-Sidak test, *p* = 0.056). **(B)** Spore enumeration was conducted for genetically modified candidates and compared to non-modified wild-type and non-toxin *C. novyi* strains. No statistical difference was determined at any time point (*n* = 3 for each strain, Holms-Sidak test, *p* = 0.058). Cell lysis was determined under both **(C)** aerobic and **(D)** anaerobic conditions by applying vegetative *C. novyi* wild-type, nontoxic, candidate a, and non-toxic candidate a to PANC-1 cells (No statistical significance was determinedby Holms-Sidak test *n* = 9 for each group, normalized to untreated controls).

Once it was established that genomic integration had occurred, each candidate was characterized to elucidate any physiological manifestation of off target gene modification events. To elicit its oncolytic effect at the most basic level, *C. novyi* must be able to sporulate and germinate as well as to lyse cells. Initially, candidates were evaluated for their ability to traverse their life cycle normally. Candidates were grown in RCM/OB broth for 72 h with aliquots harvested every 24 h. The OD_600_ of each of these candidates’ samples was observed and recorded (Figure 6A). A Holms-Sidak statistical test found no significant difference between the growth of any of the candidates and the *C. novyi* wild-type or non-toxic cells lines (*p* > 0.05). Each gene insertion candidate was then sporulated and subsequently forced to germinate back to its vegetative form. Each candidate was capable of sporulating and germinating. Additionally, upon germination, serial dilutions were used to determine colony forming units (Figure 6B). Again, a Holms-Sidak statistical test found no significant difference between any of the candidates and the *C. novyi* wild-type or non-toxic cells lines (*p* > 0.05). After the life cycle of modified *C. novyi* was established, its ability to lyse cancer cells was determined. To determine if any genes involved in the innate lytic capacity of *C. novyi* had been subjected to off-target gene modification that would lead to loss of function, *C. novyi* vegetative cells and spores were co-cultured with PANC-1 cells both anaerobically (Figure 6D) and aerobically (Figure 6C). Data presented is normalized to the no treatment in corresponding culture conditions (aerobic or anaerobic). No significant lysis occurred in any of the co-cultures when incubated aerobically and no statistical differences were determined between the genetically modified candidate A or wild-type *C. novyi* when cultured anaerobically.

## Discussion

*C. novyi* has quite the potential to become an oncotherapy—perhaps even the “holy grail” oncotherapy not only able to distinguish tumorigenic cells from normal cells but also able to do so through intravenous introduction for both primary solid tumors as well as metastases. However, the development of *C. novyi* as such a treatment is hindered at least in part by a lack of established methods with which to conduct experimentation into what makes this bacterial species so promising. Indeed, while *C. novyi*’s innate ultra-sensitivity to oxygen confers its potential to be developed as a targeted, intravenous therapy, this same characteristic is intimidating when it comes to formulating and executing experiments. Typically, the growth of anaerobic bacterial species requires expensive and cumbersome atmospheric chambers, such as gloveboxes or glovebags. Furthermore, all experimentation and equipment necessary for downstream experiments (e.g., centrifuges, vortexes, heat blocks, etc.) must also conform to these environmental restrictions, fitting inside the confines of these environmental controls. At a fundamental level, this study established the ability to work with *C. novyi* cultures outside of the restraints of a controlled atmosphere with relative ease (Figure 2), thus substantially improving both the physical and theoretical flexibility and range of methods than can be used.

Once the physical constraints of working with an anaerobic organism were eased, this study was able to adapt and build upon several other methods to increase efficacy and allow genetic manipulation of *C. novyi*, thereby further reducing the hurdles facing the clinical translation of *C. novyi* (Figure 5). One of the primary hurdles encountered is the possibility of sepsis due to off target effects. Through the addition of a 55°C incubation after the published 70°C heat cycle, alpha toxin phage DNA, responsible for much of the toxicity associate with *C. novyi*, was able to reliably and efficiently be removed (Figure 1D). After presumably increasing the safety of *C. novyi* treatments by knocking out alpha toxin, introducing new, advantageous functions via genetic manipulation became the next challenge to overcome. Previous methods had met with limited success (Cho et al., 2018; Joseph et al., 2018). According to the published efficiency rate of the original protocol (Dang et al., 2001), the modification performed here conferred an increased efficiency of approximately 10- to 100-fold (data not shown). Hence the development of methods to create and transform chemically competent *C. novyi* were overcome.

The generation of calcium competent *C. novyi* cells, demonstrated here for the first time (Figure 5B), would not have been possible without the use of RCM/OB broth to create an anaerobic environment within the broth itself, allowing *C. novyi* experimentation to occur on the benchtop. Without the ability to substantially increase transformation efficacy by approximately 100-fold and subsequently accomplish plasmid transformation within *C. novyi*, CRISPR gene editing would not have been possible. The ability to overcome low transformation efficiency gives rise to a wide range of potential further experimentation for *C. novyi*, including further attempts at genetic modification. Notably, during the process of methods optimization, it was also established that despite the high percent genetic identity shared with *C. difficile*, not all of the techniques that garner success in that particular strain can be directly applied to *C. novyi* (Figures 2B,C). This study found that though *C. difficile* demonstrates a significant response to the addition of taurocholate to growth media, particularly after germination, *C. novyi* does not. This data, combined with a lack of correlation found in Figure 3, may indicate that while *C. novyi* and *C. difficile* are cousins, *C. novyi* has many unique characteristics prohibiting the direct application of data characterizing *C. difficile*. While this study found that simple adaptations of *C. difficile* methods were effective in many cases, these protocols (i.e., sucrose gradient spore purification) had more to do with the shared physical characteristics of the two species than the biochemical or genotypic similarities (Figures 3A–E).

### Comparison to Other Bacterial Species and Techniques

The current study attempted to probe the relevance of methodologies that have been established in other bacterial species, including *C. novyi’s* closely related cousin *C. difficile* and the commonly utilized *E. coli* (Figures 3A–E) through a basic characterization of orthologous genes. Much of the current methodology available for *C. novyi* is largely based on the assumption of phenotypic similarity as a result of genotypic similarity to its more well-known cousins such as *C. difficile*. While by no means exhaustive, this study probed differences in the CAI between 3 species using the 10 most and least expressed genes from a previously published report detailing the transcriptome of *C. novyi*. The data generated in this study failed to establish any correlation between CAIs of *C. novyi*’s 10 most and least expressed genes and their corresponding orthologous genes in *C. difficile* or *E. coli*. No statistically significant differences were noted when comparing the GC skew or %R of these genes between the three species via these methods. However, a statistical significance was found for the %mol of the twenty selected genes in *C. difficile* when compared to both *C. novyi* and *E. coli*. Since this data was generated through mining published literature, it should be noted that this significance could be an artifact of the *in vitro* conditions from which this data was obtained. The current study provides a necessary glimpse into species-specific genetic differences that must be accounted for when conducting genetic modification studies such as this. As a result of this analysis, commercially available methods and algorithms based largely on model species such as *E. coli* and *C. difficile* were not solely relied upon to accomplish the generation of CRISPR elements utilized. Manual methods were included to design and verify the validity of the chosen sequences to accomplish CRISPR gene modification. Furthermore, methodology was neither excluded nor included based upon the species it had previously been successfully performed within, instead, an iterative process ensued to establish the best practices with which to accomplish genetic modification of this unique oncolytic species.

### CRISPR/Cas Gene Modification in *C. novyi*

Traditional CRISPR/Cas9 has been reported to cause increased cell death in *Clostridium* species, likely due to double strand breaks initiated by Cas9. This study was able to overcome this complication through the utilization of Cas9nickase (Cas9n)—a Cas9 with a mutated active site resulting in a single stranded DNA break encoded in the plasmid pNICKClos1.0 (Addgene). This plasmid had previously been published to accomplish gene modification in other *Clostridium* species, but not *Clostridium novyi* in particular. Thus, it remained an open question whether or not the promoters and basic genetic elements of the plasmid would work in this particular species that has demonstrated several unique characteristics when compared with other members of its genus. While a single stranded DNA nick might limit the efficacy of DNA repair, *C. novyi* experiences an innate molecular bias to homologous domain repair (HDR) rather than non-homologous end joining (Joseph et al., 2018). In theory, this bias could overcome—or at least mitigate—any decreased efficiency observed from the use of a Cas9n. When HDR occurs in response to a DNA damage event, a repair template is used that corresponds, or is homologous, to the sequences immediately flanking the breakage site (Joseph et al., 2018). The same method occurs when the DNA damage arises synthetically, such as through targeted cleavage accomplished by Cas9n. This study took advantage of this naturally occurring repair pathway to accomplish the genomic insertion of the genetic sequence encoding a simple six amino acid tag. The six amino acid tag was selected as a proof-of-concept insertion and for the purposes of this study does not serve any functional purpose beyond indicating gene editing as mediated by CRISPR/Cas9n is possible in this species of bacteria. The genetic sequence intended for insertion was prefaced by several novel elements not published in any previous bacterial CRISPR/Cas gene modification schemes: a ribosomal binding Shine-Dalgarno site as well as a TATA box promoter sequence.

While this study identified four potential gene insertion targets with two corresponding crRNA segments for each gene to accomplish the spore coat expression of a six amino acid insert, ultimately only a single target was necessary to accomplish gene editing. Generally, experimental duplicity at the molecular level is necessary with construction of the CRISPR plasmids, particularly in the context of naturally high GC content such as that seen in the *C. novyi* genome. However, surprisingly, no such complications were encountered and a single sgRNA, sgRNA002 was ultimately used for this study. This is very good news for the field because it should reduce the hesitance to attempt further gene modification studies in this particular species.

### Off-Target Effects

Currently, the field of CRISPR gene modification is just beginning to elucidate how the implementation of this particular mechanism differs in prokaryotes from that performed in eukaryotic organisms. There has been some hesitancy when attempting CRISPR gene modification in prokaryotes due to the presence of a naturally occurring CRISPR system as an equivalent of the adaptive immune system (Cho et al., 2018). This study represents one of the first to begin to probe the efficacy of such a gene modification system in a non-model bacteria. These results indicate that no measurable physiological off-target gene modification events occurred when the life cycle and lytic capacity was tested. However, it is important to note that this study was limited in that we cannot ensure expression in the spore coat based on the assays performed. Additionally, this study is further limited by the application of vegetative cells to a monolayer cell culture when it has been well-detailed that these conditions are unlikely to replicate those that are conducive for lysis *in vivo*. Yet, these studies undeniably provide necessary insights into the potential for genetically modifying oncolytic bacteria to generate better suited characteristics for clinical translation.

## Conclusion

As a unique oncolytic species, *C. novyi* has the potential to provide significant benefits to the current chemotherapeutic regimens; however, a lack of tools has hindered progress in this direction. This study described the development and modification of several key steps forward in the methodology necessary for further experimentation and commercialization of *C. novyi*. Given the exponential expansion in the applications of CRISPR/Cas mediated genomic engineering in eukaryotes to address disease states of all kinds, it is surprising that this modification has yet to gain the same level of popularity in prokaryotes. Much of the recent literature has suggested a hesitancy to attempt CRISPR-mediated gene modification in bacterial species due to the presence of endogenous CRISPR systems (Bikard et al., 2013; Barrangou, 2015; Cho et al., 2018). It is thought that any synthetic, targeted attempts to accomplish gene editing will combine synergistically to ultimately result in wide spread off-target modifications at best, and at worst systemic cell death (Bikard et al., 2013; Barrangou, 2015; Cho et al., 2018). However, as the data contained within this study demonstrates, due to the expansion of CRISPR technology, there are now ways to design an effective, selective CRISPR system despite the presence of endogenous mechanisms. In fact, we believe quite the opposite—that the presence of an endogenous CRISPR mechanism increases the on-target efficacy while decreasing the off-target capacity in ways not seen in eukaryotes.

Furthermore, much of the challenges initially encountered in applying CRISPR gene editing to bacteria can be addressed through the ever-expanding CRISPR toolkit. New alliterations on this type of genomic customization have been rapidly discovered and herald the dawn of a new era in harnessing the power of single-celled organisms. In particular, this study utilized the capacity of a Cas9 enzyme modified to create a single stranded break instead of the canonical double stranded DNA break to overcome the noted propensity that double stranded DNA breaks cause wide-spread cell death in bacteria, particularly in *Clostridial* species (Joseph et al., 2018). Special attention was taken in this study to select an originating species for the sequence corresponding to a Cas9 enzyme with a PAM sequence different from that noted for enzymes isolated from *Clostridia*, conferring an added layer of target specificity to this study. The generation of the system with which to conduct CRISPR/Cas9 gene modification contained within this report had to be applied manually due to a lack of fundamental knowledge, conferring a level of attention to detail that has been advantageously phased out with the development of commercial eukaryotic systems. It is our hope that this study will serve as inspiration leading to the generation of similar capacities in prokaryotic systems, particularly those of the oncolytic bacteria poised to become the next generation of oncotherapeutics.

## Data Availability Statement

The raw data supporting the conclusions of this article will be made available by the authors, without undue reservation.

## Author Contributions

KMD and AEB: conceptualization, writing—original draft preparation, and review and editing. KMD, RIJ, PRJ, TJW, and RJJ: data generation. KMD, AEB, and PRJ: figure creation and editing. AEB, SM, and JK: supervision and supplies. All authors contributed to the article and approved the submitted version.

## Conflict of Interest

The authors declare that the research was conducted in the absence of any commercial or financial relationships that could be construed as a potential conflict of interest.

## Footnotes

1 https://apps.thermofisher.com/crispr/index.html

2 https://www.idtdna.com/site/order/designtool/index/CRISPR_CUSTOM
